# A Data Augmentation Method for Motor Imagery EEG Signals Based on DCGAN-GP Network

**DOI:** 10.3390/brainsci14040375

**Published:** 2024-04-12

**Authors:** Xiuli Du, Xiaohui Ding, Meiling Xi, Yana Lv, Shaoming Qiu, Qingli Liu

**Affiliations:** Communication and Network Laboratory, Dalian University, Dalian 116622, China; duxiuli@dlu.edu.cn (X.D.); ximeiling@s.dlu.edu.cn (M.X.); lvyana@dlu.edu.cn (Y.L.); qiushaoming@dlu.edu.cn (S.Q.); liuqingli@dlu.edu.cn (Q.L.)

**Keywords:** motor imagery electroencephalography signals, time–frequency maps, Generative Adversarial Networks, data augmentation

## Abstract

Motor imagery electroencephalography (EEG) signals have garnered attention in brain–computer interface (BCI) research due to their potential in promoting motor rehabilitation and control. However, the limited availability of labeled data poses challenges for training robust classifiers. In this study, we propose a novel data augmentation method utilizing an improved Deep Convolutional Generative Adversarial Network with Gradient Penalty (DCGAN-GP) to address this issue. We transformed raw EEG signals into two-dimensional time–frequency maps and employed a DCGAN-GP network to generate synthetic time–frequency representations resembling real data. Validation experiments were conducted on the BCI IV 2b dataset, comparing the performance of classifiers trained with augmented and unaugmented data. Results demonstrated that classifiers trained with synthetic data exhibit enhanced robustness across multiple subjects and achieve higher classification accuracy. Our findings highlight the effectiveness of utilizing a DCGAN-GP-generated synthetic EEG data to improve classifier performance in distinguishing different motor imagery tasks. Thus, the proposed data augmentation method based on a DCGAN-GP offers a promising avenue for enhancing BCI system performance, overcoming data scarcity challenges, and bolstering classifier robustness, thereby providing substantial support for the broader adoption of BCI technology in real-world applications.

## 1. Introduction

Brain–computer interface (BCI) technology, as a key technology connecting the human brain to external devices, has demonstrated enormous potential in various fields. By capturing the electrical signals emitted by the brain, BCI systems can interpret thoughts, intentions, and perceptions, providing unprecedented possibilities for rehabilitation, entertainment, and assistive functions [1,2,3,4,5]. However, despite the broad theoretical applicability of BCI technology, in practical applications, it still faces challenges such as reliance on EEG signals [6,7] and limitations in data quality [8,9,10]. In this context, motor imagery tasks, as a commonly used BCI paradigm, have attracted widespread attention. The practice of motor imagery tasks leverages the activity patterns of participants during motor imagination to decode their intentions, thereby enhancing the understanding of the brain and improving the quality of EEG signals.

In EEG signals, different frequency components play different roles in decoding motor imagery. Generally, the μ band (8–13 Hz) and the β band (13–30 Hz) are related to motor execution and imagery. The activities in these frequency bands often exhibit event-related synchronization and desynchronization during motor imagery tasks [11] and present specific spatial patterns in the EEG, mainly concentrated in the sensorimotor cortex area [12]. Analyzing the activities in these frequency bands and specific regions can help researchers to better understand the working mechanism of the brain during motor imagery tasks, thereby improving the decoding accuracy of motor imagery intentions.

In practical applications, building and optimizing classifiers usually require a large amount of high-quality training data with more features to ensure their accuracy and robustness. EEG signal classifiers are typically based on machine learning or deep learning techniques, which require a large amount of data to learn effective feature representations. More data help the model to better capture patterns and features in EEG signals, thereby improving the performance and generalization ability of the classifier. Complex classifiers often have a large number of parameters, and insufficient training data can lead to overfitting. Increasing the amount of data can alleviate overfitting issues and improve the model’s performance on unseen data. EEG signal data may have different distribution characteristics across individuals, tasks, and experimental conditions. By increasing the amount of data, different data distributions can be better covered, making the model more adaptable. A large amount of data can enhance the stability and robustness of the model, making it more resistant to noise and interference. Such models are more reliable in practical applications. However, the collection of EEG signals [13,14,15,16] is influenced by many factors, leading to the limited availability of training data. In addition, EEG signals themselves exhibit non-stationarity and high individual variability [17,18], which limits the applicability of traditional data augmentation methods [19,20,21,22,23,24,25] such as interpolation in the brain–computer interface field.

To address these challenges, researchers have recently begun exploring the use of deep learning techniques for augmenting EEG signal data, aiming to expand limited training datasets and improve model generalization and performance. Komolovaitė et al. discussed the use of Generative Adversarial Networks (GANs) [26] and Variational Autoencoders (VAEs) to generate synthetic EEG signals. This data augmentation approach can learn data distributions and generate samples similar to real data, offering a potential solution for the brain–computer interface field. However, traditional GAN models struggle to fully capture the complexity and variability of real data, leading to issues such as mode collapse [27,28,29]. Directly applying them to EEG signal data generation poses certain challenges. To overcome this obstacle, we propose an innovative approach that combines the model structure of a Deep Convolutional Generative Adversarial Network (DCGAN) [30] with the loss function of a Wasserstein GAN with Gradient Penalty (WGAN-GP) [31]. Specifically, we transform EEG signal data into two-dimensional time–frequency maps, and then utilize the improved DCGAN model to generate time–frequency map images, thereby achieving data augmentation for EEG signal data.

**Figure 1:**
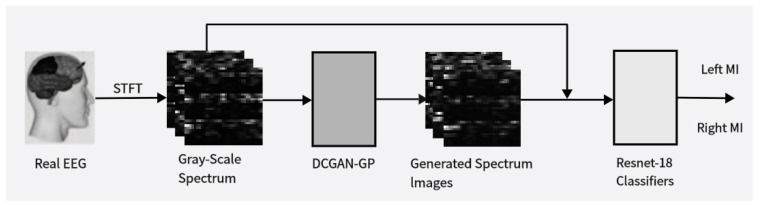
Illustrates the proposed data augmentation method. It involves using the Short-Time Fourier Transform (STFT) to obtain time-frequency images of input EEG signals. Real data is used to train the Deep Convolutional Generative Adversarial Network-Gradient Penalty (DCGAN-GP) model to generate synthetic time-frequency images. These synthetic images are then mixed with real images in proportion and used to train a convolutional classifier to distinguish between left-hand and right-hand motor imagery (MI) actions.

The objective of this study is to explore data augmentation methods for electroencephalography (EEG) signals based on deep learning techniques and assess their effectiveness in enhancing BCI classifier performance. Figure 1 provides an overview of the proposed approach in this paper; starting from the initial data collection and preprocessing, we obtained multidimensional feature maps containing both time–frequency and spatial information. These feature maps were fed into the improved DCGAN-GP model to generate corresponding image samples. We mixed these generated images with real data samples at different ratios and used the mixed data for training and evaluating our classification network. By observing changes in classification accuracy, we could infer the quality and diversity of the generated data. Based on this, we adjusted the data mixing ratios to discuss the optimal balance between real and generated data. Through comparative experiments to validate the effectiveness of the proposed method and investigate its potential applications under different conditions, we aim to offer new insights and methodologies for the further development and application of brain–computer interface (BCI) technology. By leveraging the image generation capability of the DCGAN and the advantages of the loss function in the WGAN-GP, we aim to achieve effective augmentation of EEG signal data, thereby providing more reliable support for the enhancement of BCI system performance and its practical applications.

## 2. Related Work

Traditional data augmentation methods [32] include, but are not limited to, random horizontal rotation; random adjustments of hue (H), saturation (S), and brightness (V); and random rotation, scaling, translation, and shearing. These methods generate new samples by applying various transformations and distortions to the original data, thereby increasing the diversity of the training data. However, traditional data augmentation methods have limitations [22,23,24,25]. For example, EEG signals are susceptible to noise and artifacts, making simple interpolation enhancement techniques difficult to apply effectively due to their non-stationary characteristics. Additionally, traditional methods often fail to capture the complex spatiotemporal patterns present in EEG data and the fundamental distribution of EEG signals, leading to poor results in data enhancement. Therefore, in recent years, researchers have begun to explore more advanced and effective data augmentation techniques to meet the growing demand for data and improve model performance.

Recent studies have proposed various data augmentation (DA) techniques to overcome these issues by generating synthetic EEG data similar to limited recorded signals [33]. Generative Adversarial Networks (GANs) are a promising data augmentation technique that has shown significant improvements in generating images, audio, and video data [34]. GANs can learn the potential distribution of actual data without making any assumptions and then generate synthetic samples through adversarial training between the generator network and the discriminator network. However, GAN training is typically unstable, especially in the initial stages. The loss function used in traditional Generative Adversarial Networks (GANs), which minimizes the adversarial loss between the generator and the discriminator, has some flaws: adversarial training between the generator and the discriminator can lead to oscillations and instability in the training process, sometimes even causing mode collapse, which can result in synthesized data lacking diversity and realism; also the generator may tend to learn some common patterns or features in the dataset while ignoring other important features in the dataset. This can lead to pattern bias in the generated synthetic data, which may not sufficiently represent the comprehensive features of the real data.

Abdelfattah et al. conducted one of the earliest studies to augment MI signals using GANs, where they introduced a Recursive Generative Adversarial Network (RGAN) model to generate synthetic EEG data to increase the dataset size [35]. Compared to Autoencoders (AE) and Variational Autoencoders (VAE) and their improvements [36], RGANs utilize a cyclic structure to model time-series data, which can better capture the temporal correlations in EEG data, thereby improving classification accuracy. However, an RGAN may face the issue of vanishing or exploding gradients when dealing with long sequence data, leading to training instability. Additionally, the model design overlooks the most important frequency and spatial features in motor imagery events. 

Additionally, Fahimi et al. [37,38] used Deep Convolutional Generative Adversarial Networks (DCGANs) to generate synthetic MI data, focusing on the similarity between generated data and real EEG data in both the time and frequency domains. By employing convolutional operations to extract features from input feature maps, they retained more valid information from real data, thereby increasing the diversity and authenticity of the data. However, their model improvements still led to mode collapse, where the generator becomes stuck in a local optimum, resulting in insufficient diversity in the generated samples. In [39,40], spectral images rather than the MI dataset itself were used for classification. They employed a DCGAN and a combination of a conditional VAE and a GAN to generate MI-EEG brain signals. These studies indicate that when using generated GAN data and recorded signals to train BCI systems, the accuracy of recognizing actions from EEG signals is significantly higher than when using recorded signals alone. However, issues such as gradient vanishing and training instability in the model have not been fully resolved, and there has been insufficient consideration and extraction of the multidimensional features of motor imagery EEG signals, resulting in low-quality generated samples. Zhang et al. [41] proposed a Conditional Deep Convolutional Generative Adversarial Network (CDCGAN) method aimed at improving BCI performance. This method was inspired by GANs and overcame the problem of small training datasets by automatically generating more artificial EEG signals [42]. Although this method successfully improved classification accuracy, it still did not fully cover the frequency and spatial characteristics of motor imagery signals, which may limit its effectiveness in certain applications.

Wasserstein Generative Adversarial Networks (WGANs) are a type of GAN model proposed by Gulrajani et al. [31] to address training stability issues [27,28,29]. Its innovation lies in introducing the Wasserstein distance as the distance measure between the generator and the discriminator. Compared to traditional GAN models, WGANs can more accurately evaluate the quality of generated samples, thereby avoiding mode collapse and training instability issues. However, WGANs also have some drawbacks [43], including high sensitivity to network structure and training parameters, and difficulty in handling imbalanced updates of generator and discriminator parameters.

In the study by Mao et al. [44], they proposed Least Squares Generative Adversarial Networks (LSGANs), which use the least squares loss function as the loss function of the discriminator. By minimizing the objective function of LSGANs, the Pearson χ^2^ divergence can be minimized, thereby improving the problem of gradient disappearance during training. Traditional GANs use the sigmoid cross-entropy loss function, which may lead to gradient disappearance problems. The innovation of LSGANs lies in the use of the least squares loss function, but it still does not solve other potential issues in GANs such as mode collapse. By using the loss function of the CWGAN-GP, we can further improve the training stability of GANs, thereby compensating for other potential issues that may exist in LSGANs.

In summary, while existing research has made some progress in data augmentation techniques, there are still limitations in addressing the shortage of training data and improving classifier performance. Particularly, when it comes to the frequency and spatial characteristics of motor imagery signals, existing methods have not yet achieved ideal results. Therefore, this paper explores a new method based on deep learning, proposing a novel network model, the DCGAN-GP. First, we have specifically improved the preprocessing steps to fully extract the time–frequency and spatial features of motor imagery signals, obtaining higher-quality input feature maps, which helps improve the quality and diversity of the generated data. Second, we optimized the model’s loss function by introducing the Gradient Penalty (GP) technique, reducing the risk of gradient vanishing and mode collapse during training, thereby improving the model’s performance. Finally, adaptive improvements were made to the generator and discriminator structures of the DCGAN model, enhancing the stability of the training process and speeding up the model training. The method proposed in this paper is not only applicable to the classification of EEG signals but can also be extended to other fields such as image and signal processing, demonstrating good versatility.

## 3. Model and Methods

**Figure 2:**
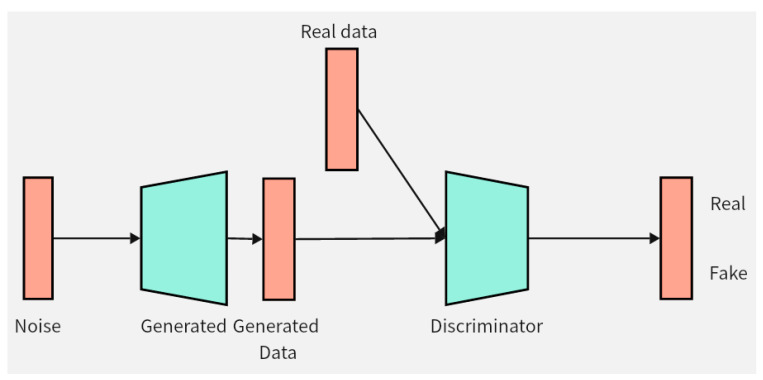
DCGAN-GP model. The green color in the figure represents the two components of the model, namely the generator and the discriminator. The orange color represents the data, and the arrows indicate the direction of data flow.

This chapter will introduce the structure and innovation of the data augmentation model DCGAN-GP proposed in this paper. Firstly, the improvement of the loss function of the DCGAN-GP will be detailed, followed by an explanation of the optimization of the model structure. Pseudocode implementation of the DCGAN-GP will also be provided to better demonstrate the implementation details of the model. Figure 2 illustrates the overall architecture of the DCGAN-GP, including the rough process of data input to the generator for synthesizing data, and then entering the discriminator for distinguishing between real and fake data.

### 3.1. Improved Loss Function

The training strategy of Generative Adversarial Networks (GANs) involves defining a game between two competing networks. The generator network maps inputs from a noise source to samples in the data space. The discriminator network receives samples from the generator or real data samples and attempts to distinguish between the two. The goal of the generator is to deceive the discriminator, while the goal of the discriminator is to accurately distinguish between these two types of samples. Formally, the game between the generator *G* and the discriminator *D* is expressed as a minimax objective function:

(1) $$\underset{G}{\min}\;\underset{D}{\max}E_{x\sim p_{data}}[\log D(x)]+E_{z\sim p_{z}}[\log(1-D(G(z)))]$$

where $p_{data}$ is the distribution of real data and $p_{z}$ is the distribution of the noise input to the generator. In ideal circumstances, by minimizing this objective function, the generator will learn to generate samples similar to the distribution of real data, while the discriminator will be unable to accurately distinguish between generated samples and real data.

However, in practice, traditional training methods can lead to problems such as gradient vanishing and mode collapse, especially as the discriminator becomes increasingly proficient. To address this issue, the Wasserstein distance has been proposed and is able to mitigate these problems to some extent. For two distributions P and Q, the Wasserstein distance is defined as the minimum cost to transform distribution P into distribution Q. In the context of GANs, this is interpreted as the minimum cost to transform the generated distribution into the distribution of real data, i.e., the minimum difference between the samples generated by the generator and real samples.

The definition of the Wasserstein distance is as follows:

(2) $$W(P_{r},P_{g})=\inf_{\gamma \in \Pi (P_{r},P_{g})}E_{(x,y)\sim \gamma }[\left\|x-y\right\|]$$

where $P_{r}$ is the distribution of real data, $P_{g}$ is the distribution of generated data by the generator, γ is a joint distribution between P and $P_{g}$ satisfying the condition that the marginal distributions are P and $P_{g}$, respectively, and $\Pi (P_{r},P_{g})$ denotes the set of all joint distributions satisfying these conditions.

The computation of the Wasserstein distance can be approximated using the Kantorovich–Rubinstein duality. For GANs, this means rewriting the loss functions of the generator and discriminator in terms of the Wasserstein distance and restricting the parameters of the discriminator to achieve this goal. This is the basic idea of the WGAN. The loss function of the WGAN changes the objective of the generator from deceiving the discriminator to maximizing the Wasserstein distance, i.e., maximizing the difference between generated samples and real samples. Its form is as follows:

(3) $$\underset{G}{\min}\;\underset{D\in D}{\max}E_{x\sim p_{r}}[D(x)]-E_{z\sim p_{z}}[D(G(z))]$$

where D denotes the set of all 1-Lipschitz continuous functions. The WGAN achieves the Lipschitz constraint on the discriminator by clipping its parameters, but this approach is often challenging to implement.

In order to better enforce the Lipschitz constraint on the discriminator, the WGAN-GP (Wasserstein GAN with Gradient Penalty) proposes an improved loss function, which adds a gradient penalty term to the original WGAN loss function. This penalty term aims to penalize the deviation of the gradient of the discriminator’s output with respect to its input. This allows for better enforcement of the Lipschitz constraint on the discriminator, thereby stabilizing the training of both the generator and discriminator. The loss functions for the generator and discriminator are as follows:

(4) $${ℒ}_{G}=-E_{z\sim P_{z}}[D(G(z))]$$

(5) $${ℒ}_{D}=E_{x\sim P_{r}}[D(x)]-E_{z\sim P_{z}}[D(G(z))]+\lambda E_{\hat{x}\sim P_{\hat{x}}}[{\left({\left\|\nabla_{\hat{x}}D(\hat{x})\right\|}_{2}-1\right)}^{2}]$$

where λ is the weight of the gradient penalty term, $\hat{x}$ is a randomly interpolated sample between real and generated data, and $P_{\hat{x}}$ is the distribution of these interpolated samples.

### 3.2. Model Architecture of DCGAN-GP

In this study, we improved and redesigned the DCGAN model, and the following are the main architecture and training details of the model.

**Figure 3:**
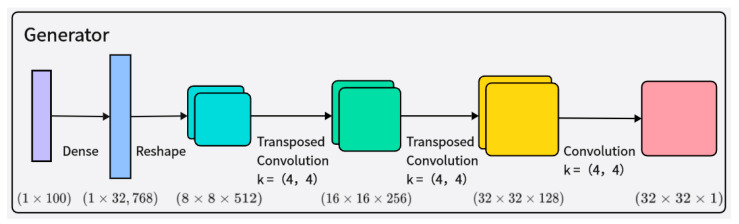
Illustration of the architecture of the generator network.

The generator G receives random noise and class labels as input, ultimately generating 32 × 32 grayscale time–frequency images. The network structure of the generator is shown in Figure 3. The generated samples are mixed with real samples corresponding to their class labels, and then fed into the discriminator D for authenticity judgment. The input to the generator is an arbitrary vector space composed of Gaussian distribution values (100 dimensions), which is then represented by a fully connected layer with a large number of nodes to generate a low-resolution version of the output image, facilitating better and faster data generation. Layer normalization is added after each transpose convolutional layer to enhance training stability. The activation function of the generator adopts the Leaky ReLU activation function.

**Figure 4:**
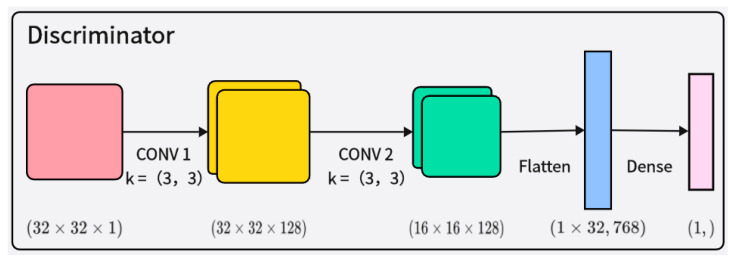
Illustration of the architecture of the discriminator network.

The discriminator D receives both real samples and samples generated by the generator and judges their authenticity, as shown in Figure 4. The discriminator’s network structure also includes layer normalization and Leaky ReLU activation functions, making the model more stable and better able to learn and generate synthetic samples that conform to the distribution of real EEG signal data. This improved architecture can effectively capture the complex features of the data, thereby enhancing the quality of the generated samples and improving training stability.

Compared to the DCGAN and WGAN-GP models, our improvements mainly focus on the structures of the generator and discriminator and the loss function. First, we have improved the generator by increasing the output size of the fully connected layers, thereby enhancing the output resolution of the generator and making the generated images clearer and more diverse. Additionally, we introduced a Reshape layer to reshape the one-dimensional vector into a high-dimensional tensor. This step not only helps to speed up the training process but also helps to retain more noise information, improving the expressive power of the generator and the diversity of the generated images.

Second, we adjusted the discriminator by redesigning the structures of the convolutional layers and fully connected layers to better adapt to the input image resolution, improving its ability to recognize real and generated images. Furthermore, we replaced batch normalization with layer normalization, which helps alleviate the problem of mode collapse during training, improving training stability and the quality of the generated images. Additionally, we introduced a gradient penalty term, further enhancing the stability and training effectiveness of the model.

In summary, our improvements mainly focus on increasing the output resolution of the generator, improving the structure of the discriminator, and introducing a gradient penalty term. These improvements make our DCGAN-GP model perform better in EEG signal data augmentation tasks, generating synthetic data that more closely aligns with the characteristics of real data, with higher quality and diversity.

During the training process, we used the Adam optimizer for stochastic gradient descent, with a momentum parameter of 0.5 and a learning rate of 0.0002. We conducted a total of 2000 iterations to train the model. The selection of this architecture and these training details aims to improve the stability of the model and the quality of generated samples, in order to better generate artificial samples that conform to the distribution of real EEG signal data.

To provide a clearer description of the training process of the proposed DCGAN-GP model in the manuscript, we present detailed pseudocode implementation below. Algorithm 1 outlines the training algorithm of the DCGAN-GP model, including the training steps for the generator G and the discriminator D, as well as the process of computing the loss function.
**Algorithm 1.** The training algorithm for DCGAN-GP.Input: Number of iterations N, discriminator D per iteration, training iterations k, batch size m, gradient penalty weight λ, discriminator update count limit $n_{critic}$Randomly initialize generator G network parameters $\theta_{G}$ and discriminator D$\;\mathrm{network}\;\mathrm{parameters}\;\theta_{G}$For i=1:N do
# Train the discriminator
4.For j=1:k do
# Collect mini-batch samples5.$\mathrm{Randomly}\;\mathrm{sample}\;m\;\mathrm{samples}\;\{z_{1},z_{2},\ldots ,z_{m}\}$ from the random noise distribution data p(z)6.Randomly sample m$\;\mathrm{real}\;\mathrm{samples}\;\{x_{1},x_{2},\ldots ,x_{m}\}$$\;\mathrm{from}\;\mathrm{the}\;\mathrm{real}\;\mathrm{distribution}\;\mathrm{dataset}\;p_{data}(x)$
# Compute the random interpolation points for the gradient penalty term.
7.Sample m$\;\mathrm{random}\;\mathrm{numbers}\;\{\lambda_{1},\lambda_{2},\ldots ,\lambda_{m}\}$ from a uniform distribution in the range [0,1]8.$\mathrm{Construct}\;\mathrm{interpolation}\;\mathrm{samples}\;{\hat{x}}_{i}=\lambda_{i}x_{i}+(1-\lambda_{i})G(z_{i})$
# Calculate the loss function of the discriminator and update the parameters
9.Calculate the loss function of the discriminator10.Calculate the gradient penalty term11.$\mathrm{The}\;\mathrm{gradient}\;\mathrm{of}\;\mathrm{the}\;\mathrm{discriminator}\;\mathrm{model}\;\mathrm{parameters}\;\mathrm{with}\;\mathrm{respect}\;\mathrm{to}\;\theta_{D}$$\;\mathrm{is}\;{ℒ}_{D}=E_{x\sim P_{r}}[D(x)]-E_{z\sim P_{z}}[D(G(z))]+\lambda E_{\hat{x}\sim P_{\hat{x}}}[{\left({\left\|\nabla_{\hat{x}}D(\hat{x})\right\|}_{2}-1\right)}^{2}]$12.End
# Train the generator
13.Collecting m$\;\mathrm{samples}\;\{z_{1},z_{2},\ldots ,z_{m}\}$ from the random noise distribution p(z)
# Calculating the generator’s loss function and updating parameters
14.Calculating the generator’s loss function.15.$\mathrm{Updating}\;\mathrm{the}\;\mathrm{generator}\;\mathrm{model}\;\mathrm{parameters}\;\theta_{G}$$\;\mathrm{with}\;\mathrm{gradient}\;\nabla_{\theta_{G}}=-\nabla_{\theta_{G}}\left[D(G(z_{i}))\right]$16.End17.Output: Generator G


## 4. Experimental Verification Design

### 4.1. Dataset Description

**Figure 5:**
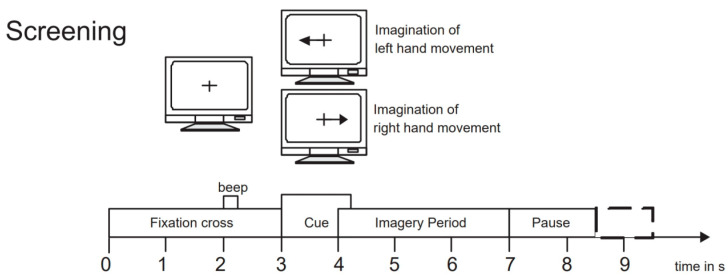
Example of the experiment process without feedback.

**Figure 6:**
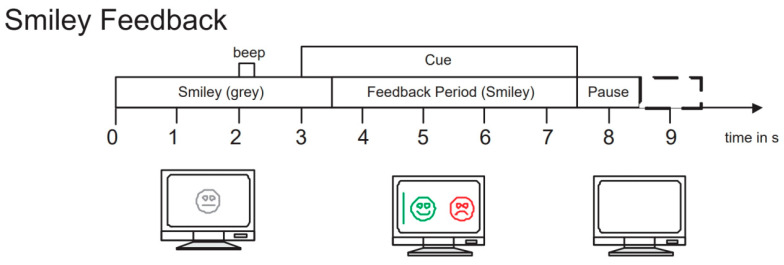
Example of the experiment process with feedback.

To evaluate the performance of our proposed network model, we selected the BCI Competition IV 2b dataset [45], which is widely used in the field of motor imagery EEG signal classification. This dataset is provided by the Graz University of Technology Brain–Computer Interface Laboratory and is specifically designed for motor imagery tasks. It includes EEG data recordings of 9 subjects performing left-hand and right-hand motor imagery tasks. During the experiment, data were collected using three electrode channels, C3, Cz, and C4, covering two motor imagery tasks (left hand and right hand). The first 3 sets of data were used for training, consisting of 400 trials of motor imagery experiments; the last 2 sets of data were used as test data, consisting of 320 trials of motor imagery experiments. The experiment process included two paradigms: no feedback and feedback. Examples of the experimental process are shown in Figure 5 and Figure 6. The first 2 sets of data each contained 120 trials without recognition result feedback; the last 3 sets of data each contained 160 trials with neural feedback of recognition results. The system would mark the screen with a green smiley face or a red frowny face according to the recognition result, indicating whether the imagined direction was correct. To ensure data accuracy, the EEG data recorded by the channels were bandpass filtered from 0.5 Hz to 100 Hz and sampled at a frequency of 250 Hz. Additionally, a 50 Hz notch filter was used to eliminate power line interference. We chose the BCI Competition IV 2b dataset primarily because it contains rich motor imagery task data and has undergone rigorous experimental design and preprocessing, ensuring the quality and applicability of the data.

### 4.2. Evaluation of the CNN Model

To evaluate our proposed GAN-based data augmentation approach, we employed two CNN classifiers for testing, namely ResNet10 and ResNet18. We compared the performance of each CNN model under two conditions: one being trained separately using the original data from each subject (i.e., non-augmented data), and the other being trained using a combination of the original data and synthetic time–frequency images generated by GAN.

The selection of neural network models plays a critical role in the performance and efficiency of deep learning systems. In our study, we chose to evaluate the ResNet-10 and ResNet-18 models due to their specific advantages and suitability for our task.

Firstly, ResNet-10 and ResNet-18 are considered lightweight models compared to deeper ResNet architectures. This characteristic is particularly advantageous in our research, as we aim to strike a balance between model complexity and computational efficiency. By opting for these models, we could achieve satisfactory performance without compromising computational resources.

Secondly, ResNet-10 and ResNet-18 have demonstrated strong performance in various image processing tasks, including image classification. Their effectiveness in handling complex data patterns while maintaining high accuracy and computational efficiency made them attractive choices for our study.

Furthermore, the ease of training and debugging of these models was a significant factor in our decision-making process. ResNet-10 and ResNet-18 are known for their ease of training, allowing us to experiment with different hyperparameters and configurations effectively.

Additionally, the wide availability of open-source implementations and pre-trained models for ResNet-10 and ResNet-18 contributed to our decision. Leveraging these existing resources facilitated the integration of these models into our research framework and enabled us to compare our results with other studies more effectively.

Another crucial aspect that influenced our choice was the residual structure of ResNet-10 and ResNet-18. The residual connections in these models enable the learning of residual mappings, which helps in training deeper networks and mitigating overfitting, especially when dealing with small datasets such as EEG signals. This characteristic was particularly relevant to our study, as we aimed to control model complexity and enhance generalization performance.

In summary, the selection of ResNet-10 and ResNet-18 was based on their lightweight nature, strong performance, ease of training, wide availability, and residual structure, which collectively contributed to the effectiveness of our EEG signal classification task.

We utilized classification accuracy as the performance metric to assess the models’ performance, calculated as the ratio of correctly classified instances. Table 1 and Table 2, respectively, present the detailed structures of the CNN models used for evaluation. Both models consist of convolutional layers, pooling layers, and fully connected layers. The convolutional layers are responsible for extracting features from input images, while the pooling layers compress data and parameters to reduce overfitting. Finally, the fully connected layers transform the output matrix into an n-dimensional vector to obtain the distribution of predictions for different classes.

### 4.3. EEG Signal Preprocessing

Research indicates that during motor imagery tasks, rhythmic brain activity occurs in the sensorimotor cortex, primarily concentrated in the mu and beta frequency bands. Therefore, we specifically focused on signal variations within these two frequency bands. In the preprocessing of EEG signals, we initially employed EEGLAB for signal processing. We selected three channels, C3, Cz, and C4, and performed independent component analysis (ICA) and signal filtering to retain frequency components ranging from 0.5 Hz to 40 Hz. These channels recorded EEG signals from the cerebral cortex.

Following signal processing, we transformed the data into grayscale spectrograms. Specifically, we utilized windows with a size of 128 points and an overlap ratio of 75%, employing 512 FFT points to achieve frequency resolution. We filtered the data within the frequency range of interest from 0.5 Hz to 40 Hz and adjusted the spectrograms of each channel to a size of 11 × 33. Finally, we vertically merged and overlaid the spectrograms of the three channels, C3, Cz, and C4, to output a spectrogram of size 32 × 32.

The purpose of these preprocessing steps is to highlight the frequency range involved in the motor imagery tasks and to preserve useful EEG signal features to the maximum extent possible. This processing approach helps improve the quality and diversity of images generated by the DCGAN-GP model, making it more suitable for applications in brain–computer interface systems.

## 5. Simulation Verification and Result Analysis

### 5.1. Image Generation Analysis

**Figure 7:**
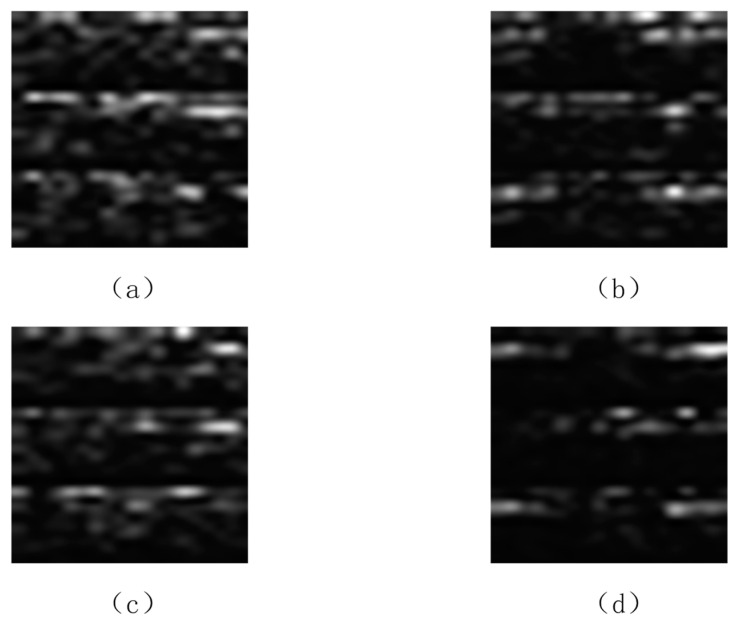
(**a**) Real sample of right-hand movement; (**b**) generated sample of right-hand movement; (**c**) real sample of left-hand movement; (**d**) generated sample of left-hand movement.

We conducted a comparative analysis between the spectrograms generated by the proposed DCGAN-GP and actual spectrograms derived from EEG recordings. Figure 7 illustrates the spectrograms corresponding to right-hand and left-hand movements from real EEG recordings, alongside the sample images generated by the DCGAN-GP network’s generator. By comparing these images, we can observe the similarities between the generated spectra and the real spectra. For instance, during right-hand motor imagery, the low-frequency mu rhythm (8–12 Hz) in the C4 channel of real samples exhibits more prolonged activity and duration throughout the event compared to the left-hand motor imagery real samples. The spectrograms generated by the DCGAN-GP model already visually present contours and contrasts similar to real samples. Despite the presence of some noticeable irregular textures and a degree of lack of naturalness in the generated samples, they closely resemble real samples in the performance of mu rhythm (8–12 Hz) and beta rhythm (13–28 Hz).

However, solely relying on visual inspection cannot comprehensively assess the quality of the generated data. To thoroughly evaluate the quality of the generated EEG spectro-temporal features, we further combined the generated samples with real samples and utilized them for neural network classification training. By observing the final classification accuracy on the real test set, we could more accurately assess the quality of the generated data.

### 5.2. Classification Result Analysis

In this section, we compare the performance of two evaluation networks trained using real data only and a combination of real and generated data. We first compare the performance when training solely with real data and then contrast it with the performance when training with a 1:1 mixture of real and generated data. Table 3 shows the classification accuracies (expressed as percentages) of different subjects under different conditions.

From individual subjects, it can be observed that the classifier’s performance is significantly improved in most cases when using the data generated by the DCGAN-GP mixed with real data. For instance, on ResNet-10, the classification accuracy for subject S1 shows the most notable improvement, increasing from 65.3% to 69.2%; similarly, subject S4 also experiences a significant improvement. Similar trends are observed on ResNet-18 as well, such as subject S5’s classification accuracy increasing from 86.9% to 90.2%, and subject S6’s classification accuracy rising from 84.6% to 87.2%. In the performance of individual subjects, some subjects exhibit better classification results after data augmentation with the DCGAN-GP, while the improvement for others is less pronounced. However, overall, the increase in average classification accuracy indicates the effectiveness of our method in improving the classification performance of brain–computer interface applications.

The ResNet-10’s average classification accuracy improved by 1.7 percentage points, and that of the ResNet-18’s by 2.5 percentage points when using the DCGAN-GP-generated data mixed with real data. Compared to using data generated by traditional DCGANs and CDCGANs, the use of the DCGAN-GP led to an average increase in classification accuracy of 1.1 and 0.1 percentage points, respectively, for ResNet-10, and 2.0 and 1.2 percentage points, respectively, for ResNet-18. These results indicate that our improved data augmentation method enhances the robustness and diversity of the models, enabling them to generate more diverse artificial samples that better match the distribution of real data, ultimately effectively improving the performance of the evaluation network. Additionally, we also observed that the performance improvement of ResNet-18 was higher than that of ResNet-10 when using our improved data augmentation method, indicating that our improvements have a more significant effect on more complex network structures. Therefore, our research results fully validate the effectiveness of our improvements to the DCGAN network in improving the quality of generated data and the performance of evaluation networks.

In summary, our improved method not only increases classification accuracy but also enhances the model’s adaptability to different samples. By introducing gradient penalty and layer normalization, we effectively alleviate the problem of mode collapse during model training and more efficiently capture the distribution characteristics of the data, thereby improving the quality and diversity of the generated data. This diversity is crucial for training robust and comprehensive models, and it helps provide valuable insights for research in brain–computer interfaces and other related fields.

### 5.3. Data Mixing Ratio and Classification Performance

In this section of the experiment, we further investigated the impact of mixing different proportions of generated samples with real samples on the classification accuracy of the ResNet-18 network. Through this series of experiments, our aim was to evaluate the effect of generated samples on classifier performance under different mixing ratios and validate the effectiveness of our proposed DCGAN-GP model in enhancing classification performance.

**Figure 8:**
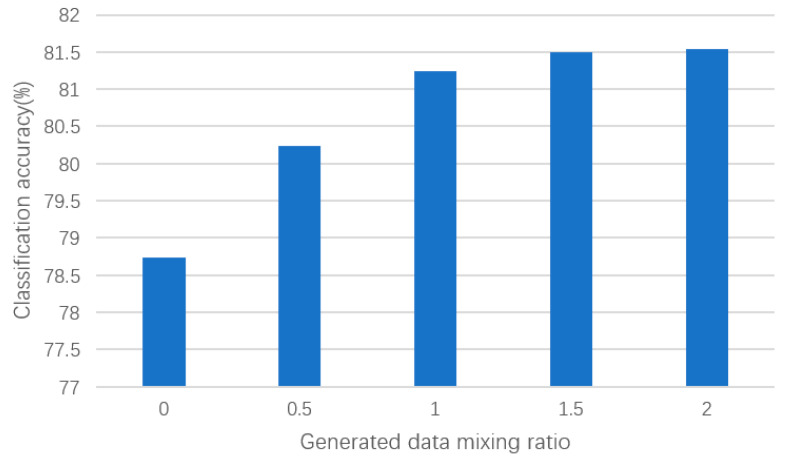
Classification performance with different proportions of generated samples.

As shown in the results of Figure 8, we observed a gradual increase in the classification accuracy of the ResNet-18 network as the proportion of generated samples increased. Specifically, when mixing 0.5 times the generated time–frequency feature samples, the classification accuracy increased by 1.49 percentage points, and when mixing 1 times the generated samples, it increased by 2.51 percentage points. This indicates that an appropriate number of generated samples can increase the diversity of the dataset, helping the classifier to better capture patterns and features in the dataset. However, when the proportion of generated samples to real samples increased to 1.5 times, the classification performance began to saturate and stabilized at around 81.4%. This may be because too many generated samples introduced too much noise, affecting the performance of the classifier. Therefore, in practical applications, a balance needs to be struck between the quantity and quality of generated samples to achieve the best classification performance.

Furthermore, our experimental results also validate the effectiveness of our improved data augmentation method in improving classification performance. Compared to data generated by traditional DCGAN networks, our improved method generates data that are closer to the distribution of real data, which is beneficial for improving the generalization ability of the classifier. This further demonstrates the DCGAN-GP model’s ability to more effectively improve the performance of EEG signal classification.

Overall, our experimental results provide important insights into the impact of data mixing ratios on classification performance, and further validate the effectiveness of our improved data augmentation method. In practical BCI applications, our research results provide valuable insights for selecting appropriate data augmentation strategies. Depending on the specific application requirements, different data mixing ratios and other data augmentation methods can be used to optimize the performance of the classifier. Future research could further explore adaptive data mixing strategies based on specific application requirements to further improve the performance and stability of BCI systems.

## 6. Conclusions

In this study, we proposed a data augmentation method based on an improved DCGAN-GP model for generating two-dimensional grayscale spectro-temporal images of EEG signals. By combining generated data with real data for training, we have demonstrated that this approach can improve the classification accuracy of EEG signals. Our main innovations include optimizing the model’s loss function and making stability improvements to the generator and discriminator structures of the DCGAN model. Through the analysis of experimental results, we found that training with generated data can enhance the performance of the evaluation network, thereby improving the accuracy of classifying EEG signals. Compared to training with only real data, mixing the datasets resulted in better classification performance across different subjects. This suggests that our proposed data augmentation method can effectively address the issue of insufficient training due to limited data in EEG signal classification tasks.

Our study also revealed several advantages of the improvements. Firstly, the introduction of generated data expands the training dataset, which helps improve the model’s generalization ability. Secondly, as the quality of generated data continues to improve, the differences between generated data and real data decrease, which helps to improve the performance of the model, especially when facing noise, interference, and individual differences in the real world. By introducing more complex loss functions or increasing data diversity, the model can become more adaptable and robust. Finally, our method is not only applicable to EEG signal classification tasks but can also be extended to other fields such as image processing and signal processing. Future directions include further optimizing model structures and training parameters, and exploring more data augmentation methods and training strategies to further enhance the quality and diversity of generated data. Future research could consider integrating EEG signals with other biosignals (such as eye movement data, electromyography signals) or non-biological signals (such as images, speech) to improve the performance and diversity of brain–computer interface systems. By designing multimodal generation models, more comprehensive and accurate data augmentation can be achieved. We believe that through continued effort and innovation, our research will make valuable contributions to the development and application of brain–computer interface technology.

## Figures and Tables

**Table 1:** ResNet-10 Network Architecture.

Layers	Filter Size	Output Dimension	Activation
Input	--	(32, 32, 1)	--
Convolution	(3, 3)	(32, 32, 64)	ReLU
Maxpool	(2, 2)	(16, 16, 64)	--
ResBlock 1	(3, 3)	(16, 16, 64)	ReLU
ResBlock 2	(3, 3)	(8, 8, 128)	ReLU
ResBlock 3	(3, 3)	(4, 4, 256)	ReLU
ResBlock 4	(3, 3)	(2, 2, 512)	ReLU
Avgpool	(2, 2)	(1, 1, 512)	--
Output (Dense)	--	2	Softmax

**Table 2:** ResNet-18 Network Architecture.

Layers	Filter Size	Output Dimension	Activation
Input	--	(32, 32, 1)	--
Convolution	(3, 3)	(32, 32, 64)	ReLU
ResBlock 1	(3, 3)	(32, 32, 64)	ReLU
ResBlock 2	(3, 3)	(32, 32, 64)	ReLU
ResBlock 3	(3, 3)	(16, 16, 128)	ReLU
ResBlock 4	(3, 3)	(16, 16, 128)	ReLU
ResBlock 5	(3, 3)	(8, 8, 256)	ReLU
ResBlock 6	(3, 3)	(8, 8, 256)	ReLU
ResBlock 7	(3, 3)	(4, 4, 512)	ReLU
ResBlock 8	(3, 3)	(4, 4, 512)	ReLU
Avgpool	(4, 4)	(1, 1, 512)	--
Output (Dense)	--	2	Softmax

**Table 3:** Comparison of Data Augmentation Results (%).

Subject	ResNet-10	ResNet-18	DCGAN/ResNet-10	DCGAN/ResNet-18	CDCGAN [37]/ResNet-10	CDCGAN [37]/ResNet-18	DCGAN-GP/ResNet-10	DCGAN-GP/ResNet-18
S1	65.3	67.9	66.7	68.4	68.3	68.9	**69.2**	**69.2**
S2	53.2	56.2	54.6	**57.2**	54.8	56.9	52.2	**57.1**
S3	64.3	65.1	64.5	64.9	64.7	65.7	63.2	**66.3**
S4	92.4	94.8	93.8	96.2	94.0	95.6	94.5	**97.9**
S5	84.5	86.9	86.2	89.3	86.4	89.3	87.1	**90.2**
S6	83.9	84.6	84.3	85.4	84.5	85.6	85.3	**87.2**
S7	78.3	79.7	78.6	80.4	79.1	80.8	79.2	**81.4**
S8	87.8	88.2	88.4	89.1	88.8	**90.1**	89.3	**90.1**
S9	85.3	87.1	86.2	86.9	85.7	87.2	86.4	**88.2**
Mean	76.7	78.7	77.3	79.2	78.3	80.0	78.4	**81.2**

## Data Availability

The data used in this study came from publicly available datasets, which can be found at the following links: https://www.bbci.de/competition/iv/ (BCI Competition IV) accessed on 22 November 2023.
